# A biological employment model of reproductive inequality

**DOI:** 10.1098/rstb.2022.0289

**Published:** 2023-08-14

**Authors:** Samuel Bowles, Peter Hammerstein

**Affiliations:** ^1^ Behavioral Sciences Program, Santa Fe Institute, Santa Fe, NM 87501, USA; ^2^ Institute for Theoretical Biology, Humboldt Universität zu Berlin, Berlin 10115, Germany

**Keywords:** reproductive skew, cooperative breeding, principal–agent model, Gini coefficient, biological markets, pay-to-stay

## Abstract

Continuing the centuries-long exchange between economics and biology, our model of reproductive skew is an adaptation of the principal–agent relationship between an employer and an employee. Inspired by the case of purple martins (*Progne subis*) and lazuli buntings (*Passerina amoena*), we model a dominant male whose fitness can be advanced not only by coercing a subordinate male but, where coercion is impossible or not cost-effective, also by providing positive fitness incentives for the subordinate that induce him to behave in ways that contribute to the dominant's fitness. We model a situation in which a dominant and subordinate contest over a variable amount of joint total fitness, both the level and division of which result from the strategies adopted by both. Thus there is not some given amount of potential fitness (or ‘pie’) that is to be divided between the two (or wasted in costly contests). The fitness incentives that in evolutionary equilibrium are conceded to the subordinate by the dominant maximize the dominant's own fitness. The reason is that the larger pie resulting from the subordinate's increased helping more than compensates for the dominant's reduced fitness share. But the conflict over fitness shares nonetheless limits the size of the pie.

This article is part of the theme issue ‘Evolutionary ecology of inequality’.

## 1. Introduction

Biology and economics alike have benefited from a robust exchange of models and metaphors. The first predator–prey model was proposed in 1786 by Joseph Townsend in his *A dissertation on the poor laws,* advocating abolition of support for the indigent [1]. The Lotka–Volterra predator–prey equations that later came to be a standard representation of the dynamics of biological systems were later borrowed by Richard Goodwin to model the macroeconomic business cycle [2]. Darwin explained that the key concept in his *On the origins of species*—’the struggle for existence’—actually came from economics: ‘the doctrine of Malthus applied … to the whole animal and vegetable kingdoms' [3, p. 63].

Darwin's own vision of evolutionary direction without intentional purpose by means of natural selection was taken up in turn by economists modelling ‘spontaneous order’ [4,5]. Notable examples are Fredrich Hayek's focus on the ‘creativity of the market’ [6] rather than individual capacities for innovation, and Gary Becker's demonstration that much of the explanatory power of economic models is due to the process of selection implemented by market competition, not to the intentionality of individual actors [7].

Continuing this fruitful interchange, the theory of biological markets—incorporating the division of labour and specialization—has illuminated forms of inter-species cooperation [8]. And John Maynard Smith's Hawk–Dove–Bourgeois game [9], which was invented to explain ‘property rights’ in such things as territories and spiders' webs, has in turn been deployed by economists to understand the evolution of private property and social norms in human economies [10,11] (S. Gulesci, S. Jindani, D. Smerdon, M. Sulaiman, E. La Ferrara, P. Young 2023, unpublished).

Here we contribute to this exchange, proposing what we term a biological employment model of reproductive skew (parallel to ‘biological markets’). We use a principal–agent model of an employer (the principal) and an employee (the agent), extended to the case of dominants and helpers in group-living non-human animals. Our model explains the use of positive incentives rather than direct coercion as a means by which a dominant may exercise power, securing help from a subordinate that he has ‘employed.’

## 2. Overview and preview

To do this we build on and extend the closely related work of Rufus Johnstone, Hanna Kokko and their coauthors [12–14]. Like them, we use economic models of bargaining to provide a synthesis of transactional and compromise (or concession) explanations of reproductive skew. Transaction models are based on the idea that a single individual controls the distribution of reproduction within a group and shares fitness with others only insofar as this is required to sustain the group, given its members' outside options [13–17].

In compromise models, by contrast, dominants do not fully control the fitness of members of their group, owing to queue-jumping [18] or when account is taken of coalitional challenges to dominants [19,20]. In these compromise (also called ‘limited control’ or ‘tug of war’) models the subordinate may obtain greater fitness than they would in their outside option [21].

Our synthesis differs from that proposed by Kokko, Johnstone & Wright [12], however, in that we address the case in which, despite being capable of limiting the fitness of other members of the group, dominants allocate greater fitness to a subordinate than is required, given the subordinate's outside options. This paradoxical voluntary fitness sharing by a dominant does not occur in their model because the dominant male is capable of ‘coercive solutions where … subordinates … are required to help the dominant’, thereby providing ‘pay-to-stay’ rent in the form of labour services [12, p. 292].

Our model provides a complement to their work, one that they mention as a possibility but do not develop, namely the use of positive incentives rather than coercion to elicit work from the helper. This might occur if the dominant's capacity to secure the subordinate's help by physical coercion were limited, as for example would be the case if it were impossible or sufficiently costly for the ‘employer’ to acquire information about the subordinate's helping activities. The conventional principal–agent model of employment shows that in this case the profit-maximizing employer will offer the employee a wage in excess of the expected value of their outside option (a spell of unemployment, searching for another job).

Similarly, in the biological employment model that follows, the dominant may concede fitness benefits to the subordinate, paying a ‘wage’ in excess of their expected fitness were they not engaged with the dominant. In our model, then, it is not the case, as Kokko and her coauthors put it, that ‘any observations of incomplete skew’ would ‘be the result of lack of dominant control over reproduction’ [12, p. 297]. The fitness benefit in excess of their outside option gained by the subordinate in our model is an incentive implemented by the dominant to maximize their own fitness, not a concession reflecting the bargaining power of the subordinate.

In the next section we introduce the idea of employment in nature, illustrated by the case of cooperative breeding. In §4 we provide a general framework encompassing both the ‘pay-to-stay’ rent that the subordinate contributes and that increases the dominant's fitness, and also the dominant's ‘wage incentive’ to the helper, which confers fitness to the helper in excess of what he would experience in the absence of his relationship to the dominant.

Section 5 illustrates the employment analogy by the relationship between dominants and subordinate helpers in purple martins (*Progne subis*) and lazuli buntings (*Passerina amoena*). In §§6 and 7 we provide a principal–agent model of this case, characterizing the distribution of fitness between the subordinate and dominant in an evolutionarily stable equilibrium. This allows us to show that by sharing fitness with the subordinate the dominant maximizes his own fitness, which is possible because the resulting increase in the joint total fitness of the two more than offsets the dominant's smaller share of the pie. In §8 we show how variations in the ecological constraints—the outside options of the two—affect the equilibrium level of reproductive skew between the two.

## 3. Employment in nature

Cooperative breeding, where one or more individuals help to raise offspring that are not their own, provides an illustration of biological employment. If a dominant individual is a prime beneficiary of such help, this individual may be in a role comparable to that of an employer who ‘hires’ or ‘fires’ its helpers by granting or denying them access to resources. The helper's ‘wage’ takes the form of direct and/or indirect (kin-related) fitness gains (superior to the helper's fitness as an isolate) that are at least partly under the dominant's control. Quite like the owner of a firm whose income typically exceeds that of their employee, the dominant animal may produce more offspring than the subordinate, and of course more so if there are multiple helpers. The resulting reproductive skew is a leading theme in the extensive literature on cooperative breeding [22,23].

Early explanations of cooperative breeding drew primarily on relatedness as a mechanism supporting helping behaviour. This is not surprising because one of most intuitive cases of helping is that of a young bird helping its parents at the nest, thereby reaping indirect fitness benefits. Yet these benefits may not be crucial for the understanding of helping [24] and, as noted by Peter Nonacs ‘kinship matters, but its main effect may be in offspring being available for manipulation while unrelated individuals are not’ [25, p. 10 163]. To focus on the more puzzling cases—helping behaviour by unrelated subordinates—we here address biological cases in which helping seems based on direct rather than indirect (kinship-related) benefits.

Ralph Bergmüller and his coauthors provide three explanations of helping behaviour through direct fitness benefits [24]. First, in ‘pay-to-stay’ explanations [26] group membership is beneficial to the subordinate but costly to the dominant, so in order for the subordinate not to be evicted it is worth his making a payment by helping the dominant. A second type of explanation invokes a helper's direct fitness benefit to other group members from ‘group augmentation’, such as the increased chance of survival when the augmented group is under attack. Third, females take helping as a costly signal of the quality of a potential mate [27]. Among these three approaches, pay-to-stay is the one that comes closest to an employer–employee relationship.

Kokko and her coauthors provided the first formal model of the pay-to-stay hypothesis [12]. In their setup, a dominant individual is in possession of a territory and a Beta individual pays a ‘rent’ to the dominant to be allowed to stay in the territory—quite like renting an apartment in the human world. The rent is measured in terms of effort put into raising offspring of the dominant. There is a reward for staying because if the dominant dies, the subordinate inherits its territory. Conceptually, this future benefit is viewed as the main reward for staying, and the analysis by Kokko *et al.* reveals conditions under which the future benefit is worth the rent.

In our—otherwise quite similar—employment model we formulate an alternative mechanism, namely the role of incentives and immediate benefits. We will show that the ‘pay-to-stay rent’ paid by the subordinate to the dominant in the Kokko *et al*. approach is part of a more general model in which a ‘wage’ is also paid by the dominant to the subordinate. To see this, before developing our model, we consider two biological examples where an older male can be viewed as employer paying a ‘wage’ to a younger male employee.

## 4. An illustration

Our first example is the socially monogamous male purple martin, *Progne subis*. In this species males occur in two visually distinct age classes. One class consists of yearlings that are sexually mature but still in immature plumage, the other class of males in adult plumage. Two parents care for the offspring, males provisioning about half of the food [28]. A single male cannot contribute enough food to support the young of two females.

Some males with adult plumage first occupy a territory, attract a first female, and mate with her. Then attracting additional females, they also tolerate a male with immature plumage, resulting in a second pair becoming tenants in Alpha's territory. The Beta-paired female then chooses to copulate with both males, and the Beta male helps her feed her offspring. Here is a description of the process:

Old males arrive at the colony early and defend extra nests. Their predawn song appears to recruit young females and males. Females are drawn to the colony by old males and young males are drawn by females. After all of the old males are paired, females pair with young males (who are permitted to occupy cavities previously defended by old males) and obtain EPCs from old males. [28, p. 181].

Young males have substantial paternity losses due to extra-pair copulations (EPCs) of their mates, whereas older males achieve almost complete paternity [29]. Since there is no indication that young males can distinguish the dominant's offspring from their own, they cannot help provisioning some of an older male's offspring when they provide care for their own brood (by-product alloparental care).

From this perspective, a yearling works as an ‘employee’ for a fully adult male. The yearling gets ‘hired’ by being tolerated to settle in close vicinity to the dominant older male's nesting site. According to Wagner and coauthors, females are not forced into copulations and show an active interest in mating with fully adult males [30].

Our second example is another passerine bird, the lazuli bunting (*Passerina amoena*). Here, brightly coloured, fully adult males defend high-quality territories and secure their dominant position by allowing only yearlings with dull female-like plumage to settle on the fringe of their territory. Delayed plumage maturation of young males may have evolved in response to such a kind of social ‘partner choice’ [8]. Greene and coauthors subjected this idea to empirical scrutiny and measured the reproductive benefits dull males have from being tolerated as neighbours at a high-quality site [31]. While these benefits were observed, it also turned out that dull males usually do not sire all their mate's offspring and thus pay a tribute to the territory holder when they care for their mixed brood. Using the terminology of Kokko and her coauthors [12], the young lazuli bunting pays to stay, quite like the young purple martin.

But in contrast to the Kokko *et al*. model, both biological employment examples have in common that the ‘payment for staying’ is not forced by physical aggression or a threat of eviction. From an evolutionary point of view, it is obvious that, conditional on being permitted to copulate with the Beta female, the Beta male need not be forced to work, provided his fitness gain through own offspring is superior to the ‘outside option’ of reproducing elsewhere.

In our bird examples the dominant male could, theoretically, increase the subordinate's parental engagement by somewhat restricting his own promiscuous tendencies. The subordinate would then sire more of his female partner's offspring and thus have an incentive to provide more care for her offspring. The principal, however, would in this case pay for the agent's enhanced ‘work ethic’ by reducing his own share of the brood the agent cares for. He would then have ‘a smaller share of a larger pie’.^1^ But will the larger pie outweigh the smaller share? As in economics, this biological principal–agent problem requires a game-theoretic treatment, which we provide in our mathematical model of biological employment.

## 5. The economics of biological employment: fitness rents and wages

To capture the main characteristics of the two biological examples just given, in the biological employment model a dominant male grants a subordinate male conditional access to a resource that may contribute to his fitness by comparison with limited fitness opportunities should he withdraw or be expelled. In economic terminology, exit from the dominance relationship means that he will suffer an unfavourable reservation (or ‘outside’) option, which is his fitness in his next best alternative, meaning, in the absence of their current interaction.

In the conventional employment model, there is a conflict of interest between the employer and the worker over the amount of work effort the worker will provide. This conflict cannot be resolved either through the use of force by the employer, as it might be with enslaved labour, or by means of an enforceable contract, as might be the case if workers could be paid by the number of units of output produced (piece rates) rather than by the hour [34,35]. But most of the tasks carried out by the employee are not susceptible to observation and measurement by the employer with sufficient precision to permit piece rate payments.

The recognition that as a result many exchanges occur even without enforceable contracts guaranteeing a given level of work done makes the economic employment model well suited to biological applications [36]. The translation from economics to biology is as follows. In the biological version of the employment model, the metric of inequality becomes the distribution of fitness between the dominant and subordinate, replacing the distribution of income (profits and wages). Access to a nesting site or other territory is the analogue to the employee's conditional access to the means of production owned by the employer. Selection on genetically transmitted variation in the strategies adopted by dominants and subordinates provides a mechanism analogous to intentional constrained optimization by employers and employees. The predicted outcomes are Nash equilibria, in which both subordinates and dominants play evolutionarily stable strategies.

We model the case in which the dominant lacks sufficient information or for some other reason cannot directly coerce the subordinate to actually provide help. As a result, the dominant offers incentives, which in this case take the form of some degree of fitness opportunities, *c*, selected to maximize the expected fitness of the dominant. We term these incentives a ‘wage’, while the helping work provided in return is what Kokko and her coauthors [12] term a ‘rent’ paid to the dominant. This feature—the dominant maximizing his fitness by conferring a fitness benefit on the agent—distinguishes this model from more a conventional setting in which what the subordinate gets is based either on his bargaining power or (if that is absent) on ‘the lowest personal fitness a subordinate will tolerate before leaving the group’ [17, p. 670].

This difference mirrors the distinction in economic modelling between two possible settings. In the first, effort is subject to contract (for example pay by piece rates) and the employer sets the least pay per unit sufficient to recruit employees (that is, not less favorable than their next best alternative.) In the second setting, a biological adaptation of which we present here, the wage must not only attract ‘employees’ but also induce them to work.

For the helper–dominant interaction to be sustained, the resulting expected fitness of the two must satisfy what economists term their respective participation constraints, as recognized (using other terminology) in the biological literature since the early work of Sandra Vehrenkamp cited above [17].^2^ The expected fitness of the subordinate, denoted *w*(*c*) because it will depend on the strategy *c* selected by the dominant, cannot be less than his reservation option, which we denote *z*. And the expected fitness of the dominant, *W*(*c*), cannot be less than his reservation option, *Z*, which is breeding without the assistance of this particular helper. (Upper-case letters for the dominant, lower-case for the subordinate.)

The conflict of interest between the two is the division of the joint total fitness of the two *W*(c) + *w*(*c*). Because the reservation options of the two are constants, we can also say that their distributional conflict is over the quantity: *W*(c) + *w*(*c*) – *z* – *Z*. Given the participation constraints, this latter expression is the ‘pie’ over which the two are contesting; both its magnitude and distribution will be determined by the dominant's choice of level of fitness permitted to the subordinate and the subordinate's response.

Does this formulation clarify how both a ‘rent’ and a ‘wage’ could be paid in the same transaction? It does. In economic terminology a rent is any payment (or here, level of fitness) in excess of one's next best alternative.^3^ So the rent received by the dominant is *W*(*c*) – *Z*, which is the contribution of the helper to the fitness of the dominant, just as in the pay-to-stay model. But the subordinate also receives a rent in economic terms equal to *w*(*c*) – *z,* which is what we termed the wage in fitness units in excess of what the helper would have had as an isolate. Both a pay-to-stay rent paid to the dominant and a wage paid by the dominant are possible if *W*(*c*) + *w*(*c*) – *z* – *Z* > 0 because the implicit cooperation between the two yields a greater total fitness than would be possible in isolation. The joint total rents of the two are what economists term the gains from cooperation or the gains from trade.

To see that these ‘wages’ and ‘rents’ characterize an evolutionary equilibrium we now present the model in mathematical terms, representing a simplification of the purple martin and lazuli bunting cases above.

## 6. The biological employment model

The dominant male provides an initial benefit to a subordinate male, namely access to the nesting site and thus the opportunity to attract a female that will be his partner for nesting and brood rearing but prone to sexually interact with both males. The model proceeds in two stages. First, the dominant and the subordinate both copulate with the subordinate's nesting partner. We assume the dominant to be in strategic control of *c*, that is, the fraction of the subordinate's brood he sires.

This includes the implicit assumption that the female—which is not explicitly depicted as a player in our game—would in equilibrium not have a net gain from further restricting the principal's genetic share of her offspring. The strong tendency of female purple martins to ‘voluntarily’ mate with dominant males supports this assumption empirically. EPCs with the dominant probably provide genetic and other benefits, such as being tolerated in a stable social environment that permits access to valuable resources, or the reduction of costs through sexual harassment. Without these advantages, it would be best for the female to only mate with the agent, whose parental effort would then be maximally induced.

In the model's second stage, we assume the subordinate has an estimate of the degree to which the offspring of his mate were sired by the dominant and that he can strategically adjust his feeding effort to this estimate.

With this stylized biological picture in mind, Alpha is first mover, choosing, *c*, the degree of Beta's sexual access to the Beta-paired female so as to maximize his own fitness, taking account of how the feeding effort that Beta will offer varies with the dominant's choice. Beta best responds to this offer; that is, he chooses the amount of food to provide to the offspring of his mate that maximizes his own fitness. The joint solution of these two fitness-optimizing problems determines the equilibrium number of copulations (Alpha's ‘wage’ offer) exchanged for an amount of care (Beta's ‘work’ effort).^4^

### (a) Beta's best (fitness-maximizing) response

The expected fitness of Beta (expressed as a percentage of the maximum biologically feasible fitness) is increasing in the amount of food provided (expressed as a fraction of the maximum feasible feeding), *f*, and decreasing in the energetic cost and predation danger of providing the food, which is associated with a mortality probability p(f) that the male will sacrifice future (mature) lifetime fitness equal to $\bar{w}$. The sequence of play is that Beta is permitted some fraction *c* of copulations with the Beta female and then provides some amount of food to his mate's offspring, at the end of the period surviving with probability 1−p(f) and gaining subsequent lifetime fitness $\bar{w}$.

To illustrate the basic intuitions of the model we provide explicit functions allowing easily derived and interpreted analytical solutions. So, we let the expected fitness of the Beta male in any period be *cf* meaning that the marginal fitness benefits of increasing feeding effort is linear in the faction of copulations that Alpha permits. (The qualitative working of the model would be unaffected if, perhaps realistically, there were diminishing marginal returns to the provision of food.) Then letting $p(f)=pf^{2}$, where *p* is a positive constant, Beta's expected lifetime fitness (this period plus the entire future) is:

$$w^{L}=cf+(1-pf^{2})\bar{w}.$$

Recognizing that *cf* is a per-period flow of additional fitness and $\bar{w}$ is a stock that can be lost by mortality, the single period contribution to Beta's fitness, *w*, is *cf* minus the hazard of forgoing future lifetime fitness^5^ or

$$w=cf-p(f)\bar{w}=cf-\bar{w}pf^{2}.$$

Beta chooses the level of feeding to maximize this fitness function by finding the value of *f* that sets to zero the derivative of *w* with respect to *f*, that is

$$w_{f}=c-2\bar{w}pf=0.$$

This means that Beta selects the level of feeding such that the marginal benefit of feeding, namely *c,* equals the marginal cost of feeding, $2\bar{w}pf$. Notice that an increase in mortality risk or expected future fitness will increase the marginal cost of providing food. We thus have the fitness-maximizing level of feeding, Beta's strategy in the game:

$$f^{*}(c)=\frac{c}{2\bar{w}p}.$$

This is Beta's best response function (also termed the *incentive compatibility constraint*) limiting the principal (the * indicating the solution to the relevant fitness-maximizing problem). It expresses the primary mechanism in the model: that Alpha, by allowing Beta more copulations, raises the marginal fitness benefit to Beta of providing more food to the Beta female, which contributes to Alpha's fitness (unless *c* = 0, i.e. the Alpha has ‘assigned’ all of the copulations to the Beta).

### (b) Alpha's fitness-maximizing ‘wage’ offer

The contribution of the Beta couple to Alpha's per period fitness ($W^{\beta }$) is just his share of Beta's surviving offspring, which, taking account of Beta's best response function, can be written:

$$W^{\beta }=(1-c)f^{*}=(1-c)\frac{c}{2\bar{w}p}.$$

Alpha then chooses *c* to maximize this fitness function by differentiating $W^{\beta }$ with respect to *c* and finding the value of *c* that sets the result equal to zero:

$$W_{c}^{\beta }=\frac{1-c}{2\bar{w}p}-\frac{c}{2\bar{w}p}=0\Rightarrow c^{*}=\frac{1}{2}.$$

As in the case of Beta (above), this requires that Alpha allow Beta a fraction of copulations such that the marginal benefit to Alpha (due to Beta's increased feeding of his mate's offspring, some of which carry Alpha's genes, the first term) is equal to the marginal cost (due to copulations allowed to Beta, the ‘fitness-sharing’ second term).

What this means is that, for *c* < ½, granting the Beta greater opportunities to copulate with his own mate is a fitness-maximizing strategy for the dominant because it increases the size of the total fitness pie by proportionally more than it reduces the dominant's share of the pie. For *c* > ½, however, the reverse is true: further concessions also increase the size of the pie, but they reduce the dominant's share by more than they increase the pie. In this case the dominant prefers a larger share (that is ½) of a smaller pie.^6^

## 7. Fitness inequality in an evolutionarily stable biological employment transaction

Using the results of the fitness-optimizing processes of both Beta and Alpha and the choice of the Beta female, we find that the amount of food provided by Beta is:

$$f^{*}=\frac{c^{*}}{2\bar{w}p}=\frac{1}{4\bar{w}p},$$

so, recalling that c* = ½ and using this best response in Beta's fitness function, we have:

$$w^{*}=\frac{1}{2}\frac{1}{4\bar{w}p}-\bar{w}p{\left(\frac{1}{4\bar{w}p}\right)}^{2}=\frac{1}{8\bar{w}p}-\frac{1}{16\bar{w}p}=\frac{1}{16\bar{w}p}.$$

Beta's contribution to Alpha's fitness is:

$$W^{\beta^{*}}=(1-c^{*})f^{*}=\frac{1}{8\bar{w}p},$$

which is twice Beta's fitness because Alpha does not endure the mortality risk of feeding the offspring of the Beta female.

To determine the total fitness of the Alpha, recall that the Alpha-paired female does not engage in EPCs.^7^ If his fitness function is identical to Beta's except that there is no equivalent of *c,* then, denoting the level of the Alpha male's contribution to feeding his own young as *F*, Alpha's ‘own mate’ contribution to his fitness will be:

$$W^{\alpha *}=F-\bar{w}pF^{2}.$$

Like Beta, Alpha will select *F* to maximize this function setting

$$W_{F}^{\alpha *}=1-2\bar{w}pF=0\Rightarrow F^{*}=\frac{1}{2\bar{w}p},$$

which is the same level of food provision that Beta would provide to the offspring of the Beta female, were Alpha to engage in no EPCs with the Beta female (that is, so that *c* = 1). Using Alpha's fitness-maximizing value, F*, we have the contribution of the dominant-paired female to Alpha's fitness:

$$W^{\alpha *}=\frac{1}{2\bar{w}p}-\bar{w}p{\left(\frac{1}{2\bar{w}p}\right)}^{2}=\frac{1}{4\bar{w}p}.$$

Finally, adding this to the fitness contribution of the Beta couple derived above, the total per-period fitness of Alpha will be:

$$W^{*}=W^{\beta *}+W^{\alpha *}=\frac{3}{8\bar{w}p}.$$

### (a) An unequal and inefficient evolutionary equilibrium

The Gini coefficient for the two with the above fitnesses is 0.71 (similar to wealth inequality in many modern economies).^8^ The above set of values [c*, f*, F*] generating the resulting distribution of fitness between Alpha and Beta constitute a Nash equilibrium, meaning that, given the strategy adopted by the other, neither Alpha nor Beta could have higher fitness were they to adopt a different strategy. This is the case because the terms of the exchange are the result of both parties fitness being maximized, given the strategy adopted by the other.^9^ The outcome, then, is self-enforcing; there is no agreement (or ‘contract’) on which one or the other can renege.

An implication is that the outcome [c*, f*, F*] is evolutionarily stable in the sense that these strategies yield higher fitnesses for each of the two than would any ‘invading’ (by mutation or migration) alternative strategy by the other. So, a mutant would-be Beta, otherwise identical to the one modelled here, could not outcompete the incumbent Beta by ‘offering’ the same feeding services in return for a reduced level of copulations, as this offer (if accepted) would result in a lower level of fitness.

While evolutionarily stable, the equilibrium allocation is not Pareto-efficient, meaning that at least one of the two males could have greater fitness and the other not less in some alternative technically feasible allocation. An example of such a Pareto superior alternative allocation in which both would have greater fitness is that Alpha permitted slightly more than c* copulations to Beta and Beta provided slightly more food than f*. Where they able to ‘agree’ to an ‘enforceable contract’ specifying this, their fitness-maximizing choice would be to both ‘sign’.^10^

But in biology there are no such contracts to be signed, so not surprisingly Pareto-inefficient outcomes are to be expected in strategic interactions [36]. Even in economics, the principal–agent model of employment illustrates the case where there are limits, too, to the kind of contracts that can be written and enforced among humans—with the same result.

Given the conflict of interest between the subordinate and the dominant, the strategies adopted in pursuit of individual fitness advantage result in the joint total fitness of the two being more than had Alpha monopolized all of the copulations with the Beta female. But given the inefficiency of the resulting allocation just demonstrated, it is no surprise that the total joint fitness is less than the maximum feasible. To see this, write the fitness of the two as:

$$\mathrm{joint}\;\mathrm{total}\;\mathrm{fitness}=w+W^{\beta }+W^{\alpha }=\frac{c^{2}}{4\bar{w}p}+\frac{c(1-c)}{2\bar{w}p}+\frac{1}{4\bar{w}p}.$$

So, instead of the equilibrium value of *c* = ½, the maximum total fitness would result if Alpha were to forgo any EPCs with the Beta female (*c* = 1). Apart from not being implementable by the adoption of fitness-maximizing strategies, this outcome and the resulting Gini coefficient—namely, zero, the absence of reproductive skew—would deprive Alpha of any fitness advantage of allowing Beta access to the territory.

## 8. Ecological constraints

To address the limits of the bargaining outcomes set by the participation constraints of the two, we now impose two requirements for an evolutionarily sustainable outcome. First, the expected fitness of the Beta male (and mate) must be sufficient to attract them to Alpha's site. And second, the contribution of the Beta pair to the fitness of Alpha must also be sufficient that Alpha gains fitness by granting them access to the site. Taking account of the quality of alternative sites and mating opportunities, including predation risks and other costs of dispersal, if Alpha were not to grant Beta access to the site, Beta would have some given level of expected fitness, *z*. To attract Beta, then, Alpha's ‘offer’ would need to bear an expected fitness for Beta not less than *z,* or:

$$w=cf-\bar{w}pf^{2}=\frac{c^{2}}{4\bar{w}p}\geq z.$$

This is Beta's participation constraint*,* limiting the offers that Alpha can implement. We rearrange and rewrite this equation as an equality, giving the least fraction of copulations offered to Beta sufficient to attract him to Alpha's site:

$${\bar{c}}^{2}=4\bar{w}pz\;\mathrm{or}\;\bar{c}={\left(4\bar{w}pz\right)}^{\frac{1}{2}}.$$

There is a corresponding maximum value of *z* such that satisfying Beta's participation constraint would require allowing Beta to have all of the copulations with his mate (*c* = 1) and thereby eliminating any fitness benefit that Alpha would obtain by engaging the help of Beta.

This then gives us a complete statement of the fitness-maximizing strategy of Alpha, with the first condition below relevant for the range of *z* such that the incentive compatibility constraint (Beta’s best response function) is binding, and the second for the range such that the participation constraint is binding:^11^

$$\;\;\;\begin{array}{ccc} c=\frac{1}{2}\; & \mathrm{if}\;z\leq \frac{1}{16\bar{w}p}, & \;\mathrm{so}\;\mathrm{Bet}a^{\prime }s\;\mathrm{incentive}\;\mathrm{compatibility}\;\mathrm{constraint}\;\mathrm{is}\;\mathrm{binding};\\ c=\bar{c}={\left(4\bar{w}pz\right)}^{\frac{1}{2}} & \mathrm{if}\;z>\frac{1}{16\bar{w}p}, & \;\mathrm{so}\;\mathrm{Bet}a^{\prime }s\;\mathrm{participation}\;\mathrm{constraint}\;\mathrm{is}\;\mathrm{binding}.\; \end{array}$$

If, however, $z\geq 1/4\bar{w}p$, there is no way that Alpha can benefit from an offer that would can satisfiy Beta's participation constraint. In this case the would-be Beta has an alternative fitness such that the minimal acceptable offer, in the case that the above equation is satisfied as an equality, would be *c* = 1, so Beta would not contribute to Alpha's fitness at all.

Taking account of the ecological setting in which the interaction takes place, the Gini coefficient measuring fitness inequality between Alpha and Beta ranges from 0.71, if the incentive compatibility constraint is binding, to zero (complete equality) as *z* rises to the maximum limiting value given by the above equations. Thus, as has been shown by Vehrencamp and many others, more favorable outside options for the subordinate reduce reproductive skew over the range of values of *z* such that the participation constraint is binding [17].

The reason is that, over this range, increases in *z* reduce the fitness difference between the two and at the same time increase the total fitness. The latter is because increases in *z* force the dominant to allow Beta more copulations in order to attract him and (as seen above) total fitness is increasing in *c*. The effect is to reduce the fitness difference relative to the average fitness, which is what the Gini coefficient measures.

Over this same range, an increase in the cost of providing food (a higher predation risk, *p* or expected adult lifetime fitness, $\bar{w}$) reduces the Gini coefficient. This is because, by shifting the incentive compatibility constraint (adversely from Alpha's standpoint), these changes reduce the amount of food that Beta will provide for any level of copulations allowed by Alpha, effectively increasing the cost to Alpha of acquiring the services of the helper.

## 9. Discussion: conflict over fitness shares reduces the size of the fitness ‘pie’

Stimulated by the case of two cooperatively breeding socially monogamous species we have represented the relationship of a dominant to a subordinate in a principal–agent model and derived expressions for the resulting degree of reproductive skew. Though inspired by these two cases of cooperative breeding, the model is in essence applicable to any species in which subordinates contribute to the fitness of dominants through coalitional support or other helping activity that the dominant cannot directly control. Many additional aspects may, of course, need consideration (e.g. negative effects on Alpha through local food depletion, or positive effects through predator detection or joint defence). We have shown that fitness sharing beyond that required by the subordinate's outside options, even by a dominant that is capable of regulating the fitness of a subordinate, may be evolutionarily stable.

But the equilibrium level of fitness sharing by the principal is less than would maximize the joint fitness of the two. Our model thus provides another case in which, citing Kokko, ‘there is a way for a genotype to succeed disproportionately by exploiting conspecifics' [38, p. 5] so that ‘adaptation can lead to declining reproductive output of a population’ [38, p. 1].^12^ An especially transparent example from evolutionary biology is Maynard Smith's Hawk–Dove game, in which population mean fitness is maximized if Hawks are entirely absent, but the Dove strategy is not evolutionarily stable, so the all-Dove population will be invaded by Hawks [9].

As in the principal–agent relationships studied here, it will generally be the case that the size of the total fitness pie over which conflicts occur will be affected by the strategies adopted by individuals to increase their share of the pie. Where there exist individual fitness-maximizing strategies that are costly in the sense that they reduce the size of the pie but individually advantageous (they increase the actor's own slice of the pie, as for example when the dominant grants only *c* = ½ to the subordinate), the result may be a smaller equilibrium size of the pie.

Examples from human conflicts include the substantial number of people employed as what is termed ‘guard labour’, whose task is not to produce goods but to prevent their being stolen or to discipline employees (maintaining the pace of work, for example), or to increase a company's market share at the expense of other firms by means of advertising [39,40]. Further examples of pie-reducing, share-increasing strategies are strikes for higher wages and other cases similar to the war of attrition game. It is readily shown (under plausible conditions) that in the symmetric version of this game, the costs borne by the players exhaust the ‘pie’ over which they are contesting, entirely canceling the potential gains from cooperation in the underlying game [41].

This brings us back to the ongoing creative interchange between economics and biology to which we hope to have contributed. The war of attrition, borrowed from First World War military terminology, was first mathematically modelled by theoretical biologists John Maynard Smith [9] and D. T. Bishop & C. Cannings [42], representing conflicts among non-human animals.^13^ The same model has subsequently been applied to economic conflicts among humans, not only to strikes but also to pricing strategies in auctions and the competitive bribery of corrupt officials.

## Data Availability

This article has no additional data.

## Footnotes

1 A structurally similar problem, not relating to employment, has been highlighted by Galipaud & Kokko [32]. Jointly nesting females, which all lay their eggs into one nest, may remove each others' eggs to have—in this case—a ‘larger share of a smaller pie’. The ‘egg-tossing’ behaviour in communal guira cuckoos (*Guira guira*) lends empirical support to this idea [33].

2 In Kokko *et al.* [12], exact equivalents of the following participation constraints appear as equation 3a and 3b for the case where the interactions are among non-kin (that is with *r* = 0).

3 Though our models are equivalent in this respect, we differ in that we measure the object of conflict—the pie—as the total rents, while Kokko *et al.* [12] measure it as the level of work provided by the helper, ranging from a low level such that the dominant would be no better off fitness-wise than in the absence of the helper (and therefore receiving no rent) to a high level of help such that the subordinate's fitness would not exceed his reservation option (with the subordinate therefore receiving no rent).

4 Alpha need not control the *number* of copulations by Beta with his mate. What matters in the model is (a) that he can regulate *c,* Beta's share of copulations, by the frequency with which he (Alpha) copulates with her, and (b) that Alpha cannot directly control the feeding effort of Beta.

5 In economic terminology *cf* is equivalent to gross income per period, while $\bar{w}pf^{2}$ is expected depreciation of the stock of wealth in a given period; the difference between the two is analogous to income properly measured (sometimes termed net income).

6 For young purple martin males, Wagner *et al.* [30] find an average paternity of the brood they raise of about 40%, which coincidentally is close to our equilibrium value of *c**. But our *c** = ½ is not derived from empirical data on any particular species; the value is model-specific, serving as an illustration of the model's working. A quantitative study of purple martin paternity distribution would, of course, require a model incorporating more biological detail about the species and its ecology.

7 Doing so would reduce her expected fitness, as it might reduce the care of her young provided by Alpha and there might be genetic disadvantages as well.

8 The Gini coefficient is one-half the mean difference in fitness between all unique pairs in the population relative to the mean fitness, which in this case reduces to 5/16 divided by 7/16, or 5/7 = 0.71. On what the Gini coefficient measures, see [37].

9 The strategy adopted by Beta is his best response function, not any particular level of *f,* which depends on Alpha's strategy (choice of *c*).

10 The existence of this so-called Pareto-superior alternative allocation is guaranteed by the fact that from the first-order conditions respectively for Beta's choice of *f* and Alpha's choice of *c* we know that both $w_{f}=0$ and $W_{c}^{\beta }=0$, so there must be some small increases both in *f* (conferring benefits on Alpha) and in *c* (conferring benefits on Beta) that would simultaneously increase the fitness of both.

11 A constraint is said to be binding when it affects the behaviour of the actor. When the incentive compatibility constraint is binding, Alpha maximizes his fitness by offering a level of copulations to Beta that provides a fitness rent, and hence is more than sufficient to attract him and his mate to the site (so the participation constraint is not binding, that is to say it is redundant in the sense that altering it by a small amount would not affect the actors' behaviours).

12 Kokko notes that cases of this type are ‘particularly readily observable when males participate in a zero-sum game’ [38]. Our model provides a transparent case where the same result occurs in a non-zero-sum setting.

13 As far as we are aware it was Bishop & Cannings and Maynard Smith (respectively), who first pointed out that ‘the energy expended in the conflicts … exactly balances the energy obtained from the rewards' and that the bargaining [42, p. 112] strategy that is ‘the ESS is not that which would be favoured by group selection’ [9, p. 215].
